# On prime powers in linear recurrence sequences

**DOI:** 10.1007/s40316-021-00163-9

**Published:** 2021-04-18

**Authors:** Japhet Odjoumani, Volker Ziegler

**Affiliations:** 1 Institut de Mathématiques et de Sciences Physiques, Université d’Abomey-Calavi, Dangbo, Benin; 2 University of Salzburg, Hellbrunnerstrasse 34/I, 5020 Salzburg, Austria

**Keywords:** Diophantine equations, Linear recurrence sequences, Exponential Diophantine equations, 11D45, 11B37, 11D61

## Abstract

In this paper we consider the Diophantine equation $U_n=p^x$ where $U_n$ is a linear recurrence sequence, *p* is a prime number, and *x* is a positive integer. Under some technical hypotheses on $U_n$, we show that, for any *p* outside of an effectively computable finite set of prime numbers, there exists at most one solution (*n*, *x*) to that Diophantine equation. We compute this exceptional set for the Tribonacci sequence and for the Lucas sequence plus one.

## Résumé

Nous considérons dans cet article l’équation $U_n=p^x$, où $U_n$ est une suite récurrente linéaire, *p* un nombre premier, et *x* un entier positif. Sous des hypothèses techniques, nous montrons que, pour tout *p* en dehors d’un ensemble fini calculable de nombres premiers, cette équation admet au plus une solution (*n*, *x*). Nous déterminons cet ensemble exceptionnel pour la suite de Tribonacci et pour la suite de Lucas plus un.

## Introduction

Let $U=(U_n)_{n\ge 0}$ be a sequence of integers satisfying a recurrence relation

$$\begin{aligned} U_{n+d}=s_{d-1}U_{n+d-1}+\dots +s_0U_n, \quad n\ge 0. \end{aligned}$$

Let us assume that this linear recurrence is of order *d*, that is $U_n$ does not satisfy a recurrence relation for a smaller *d*. We denote by

$$\begin{aligned} p_U(X)=X^d-s_{d-1}X^{d-1}-\dots -s_0=\prod _{i=1}^t \left( X-\alpha _i\right) ^{m_i} \end{aligned}$$

its characteristic polynomial. In case that all multiplicities $m_i$ are equal to 1 we call *U* a simple recurrence relation. If *U* is a simple recurrence sequence of order *d* we can write

$$\begin{aligned} U_n=a_1\alpha _1^n+a_2\alpha _2^n+\dots +a_d\alpha _d^n \end{aligned}$$

with $a_1,\dots ,a_d\in {\mathbb {C}}{\setminus }\{0\}$. If the characteristic roots also satisfy

$$\begin{aligned} |\alpha _1|>|\alpha _2|\ge \dots \ge |\alpha _d| \end{aligned}$$

we say that *U* satisfies a dominant root condition. If we even have

$$\begin{aligned} |\alpha _1|>|\alpha _2|>|\alpha _3|\ge \dots \ge |\alpha _d| \end{aligned}$$

we say that *U* satisfies a strong dominant root condition. We say that *U* satisfies the multiplicative independence condition if the numbers $a_1$ and $\alpha _1$ are multiplicatively independent, that is that the only integral solution to $a_1^x\alpha _1^y=1$ is $(x,y)=(0,0)$.

A sequence of special interest in this paper will be the Tribonacci sequence $T=(T_n)_{n\ge 0}$ which is a linear recurrence sequence of order 3 given by

$$\begin{aligned} T_{n+3}=T_{n+2}+T_{n+1}+T_n,\quad n\ge 0 \end{aligned}$$

and $T_0=0$, $T_1=1$ and $T_2=1$. We will also have a closer look on the linear recurrence sequence $L=(L_n)_{n\ge 0}$ also of order 3 given by

$$\begin{aligned} L_{n+3}=2L_{n+2}-L_n,\quad n\ge 0 \end{aligned}$$

and $L_0=3$, $L_1=2$ and $L_2=4$. Note that this recurrence sequence is the classical Lucas sequence ${\tilde{L}}=({\tilde{L}}_n)_{n\ge 0}$ added by 1, where ${\tilde{L}}$ is given by

$$\begin{aligned} {\tilde{L}}_{n+2}={\tilde{L}}_{n+1}+{\tilde{L}}_n, \quad n\ge 0 \end{aligned}$$

and ${\tilde{L}}_0=2$ and ${\tilde{L}}_1=1$. In particular, we have $L_n={\tilde{L}}_n +1$ and will therefore call *L* the added by one Lucas sequence.

There is a rich literature on Diophantine equations involving linear recurrence sequences and we refer to [7] for an overview. In this paper we will focus on Diophantine equations of the form $U_n=y^m$. In the case that $U_n$ is a binary recurrence sequence Pethő [11] and, independently, Shorey and Stewart [12] (see also [13, Chapter 9]) showed that such equations have only finitely many solutions $(n,m,y)\in {\mathbb {N}}^2 \times {\mathbb {Z}}$ with $y,m>1$. In the case of higher order recurrence sequences Shorey and Stewart [12] showed that under a dominant root assumption a solution (*n*, *y*, *m*) to $U_n=y^m$ with $y,m>1$ satisfies $m<C$, where *C* is an effectively computable constant. Moreover, a recent result due to Bugeaud and Kaneko [6] implies that the equation $U_n=y^m$ with $y,m>1$ has at most finitely many solutions and their number can be explicitly determined.

However, for a given binary sequence $U_n$ it is a very hard task to find all solutions to $U_n=y^m$. In the case of the Fibonacci and the Lucas sequence this task was finally resolved by Bugeaud, Mignotte and Siksek [5].

In the case of recurrence sequences of higher order (satisfying a dominant root condition) the methods fail to find all solutions (*n*, *y*, *m*) to $U_n=y^m$ with $y,m>1$ and results similar to those of Pethő, Shorey and Stewart for the binary case have not been proved yet.

In this paper we are interested in the following problem. Let $U=(U_n)_{n\ge 0}$ be a fixed sequence and ${\mathcal {P}}=\{p_1,\dots ,p_t\}$ a finite set of primes. How many solutions $(n,x_1,\dots ,x_t)$, with $n,x_1,\dots ,x_t$ non-negative integers does the equation

$$\begin{aligned} U_n=\pm p_1^{x_1}\cdots p_t^{x_t} \end{aligned}$$

have? Of course the theory of *S*-unit equations yields a bound for the number of solutions depending only on *t*. These bounds are usually far from the optimal bound and do not reflect what can be expected from numerical experiments. In this paper we resolve the case that $t=1$. That is we consider Diophantine equations of the form $U_n=p^x$ for a fixed sequence $U=(U_n)_{n\ge 0}$ and arbitrary, but fixed prime *p*. By Baker’s method using lower bounds for linear forms in logarithms this equation can be solved easily for particular values for *p*. However, in this paper we show that up to a finite effective computable set of primes $S=S(U)$ the Diophantine equation $U_n=p^x$ has at most one solution. More precisely we show:

### Theorem 1

Let $U=(U_n)_{n\ge 0}$ be a simple, linear recurrence sequence of order at least two, satisfying the dominant root condition. Further we assume that $U_n$ satisfies one of the following conditions

- *U* satisfies the strong dominant root condition;
- *U* satisfies the multiplicative independence condition.

Then there exists an effective computable set of primes *S* such that the equation $U_n=\pm p^x$ has at most one solution $(n,x)\in {\mathbb {N}}^2$ with $x\ne 0$ unless $p\in S$.

### Remark 1

Let us note that the assumption that *U* is simple can be dropped. To avoid technical difficulties we refrain from this extension.

The set *S* in Theorem 1 is not only effective computable, but we can also provide an efficient algorithm to compute the set *S* and find all exceptional primes. We demonstrate the method by applying it to the Tribonacci sequence *T* and the sequence *L* and prove the following two theorems:

### Theorem 2

The Diophantine equation $T_n=p^x$ has at most one solution (*n*, *x*) with $x\ne 0$ unless $p=2$. In the case that $p=2$ there exist two solutions, namely $T_3=2$ and $T_4=4$.

### Theorem 3

The Diophantine equation $L_n=p^x$ has at most one solution (*n*, *x*) with $x\ne 0$ unless $p=2$. In the case that $p=2$ there exist three solutions, namely $L_1=2$, $L_2=4$ and $L_4=8$.

It is easy to see that the Tribonacci sequence *T* is indeed of order three, is simple and satisfies the dominant root condition but not the strong dominant root condition. However, the Tribonacci sequence satisfies the multiplicative independence condition (see also Sect. 2).

On the other hand it is easy to see that the Lucas sequence added by 1, i.e. the sequence *L*, is simple and of order three and satisfies the strong dominant root condition, but does not satisfy the multiplicative independence condition. Moreover let us note that a result of Shorey and Stewart [12, Theorem 4] implies that the Diophantine equation $L_n=y^m$ has only finitely many solutions (*n*, *y*, *m*) with $y,m>1$ and *n* even. This implies even a stronger result than stated in Theorem 3, considering only even indices *n*. But to our knowledge no such result is known for odd indices *n* and thus our result (Theorem 3) indeed complements the result due to Shorey and Stewart.

Finally let us note that Theorem 1 can be easily proved in the case that *U* is a Lucas–Lehmer sequence, by applying the Primitive Divisor Theorem proved by Bilu, Hanrot, and Voutier [4]. Therefore we choose the sequences *T* and *L* to avoid cases that could easily be solved by the Primitive Divisor Theorem. We also want to note that the result of Theorem 2 can easily be deduced by a result due to Gomez and Luca [8] who found all multiplicatively dependent pairs of *k*-generalized Fibonacci numbers. However, let us note that our proof of Theorem 2 yields an alternative approach.

In the next section we provide some useful lemmas and inequalities. In Sect. 3 we will use Baker’s method to obtain upper bounds for *n* in the case that *p* is arbitrary but fixed and will therefore reprove a theorem due to Shorey and Tijdeman [12, Theorem 3]. The heart of the proof is in Sect. 4, where we show that the existence of two solutions $(n_1,x_1)$ and $(n_2,x_2)$ implies an effectively computable upper bound for $n_1$. This allows us to finally prove Theorem 1. Unfortunately the absolute upper bounds are too large to successfully perform a computer search. However, in the case of the Tribonacci sequence and the Lucas sequence added by one the bound for $n_1$ is small enough to find small lists of possible candidates of primes *p* such that the Diophantine equations $T_n=p^x$ and $L_n=p^x$ may have two solutions. Using the upper bounds found in Sect. 3 together with the Baker–Davenport reduction (see Lemma 6) and using lower bounds for approximations by continued fractions (see Lemma 5) we find all primes *p* that admit more than one solution. These reduction procedures are discussed in the final Sect. 5.

Throughout the paper we will denote by $C_1,C_2,\dots$ positive and effectively computable constants.

## Auxiliary results

To ease the notation let us write $a=a_1$ and $\alpha =\alpha _1$. Since $U_n$ is defined over the integers all roots of the characteristic polynomial are algebraic integers. Since $U_n$ is simple, of order at least two and satisfies a dominant root condition we conclude that $|\alpha |>1$ and furthermore that *a* and $\alpha$ are real. Replacing $U_n$ by $(-1)^nU_n$ or $(-1)^{n+1}U_n$ or $-U_n$ we may also assume that both $\alpha$ and *a* are positive.

With these assumptions and notations in force there exist effective computable constants $C_1,C_2,C_3>0$ such that

1 $$\begin{aligned} C_1 a\alpha ^n<U_n=p^x<C_2 a\alpha ^n \end{aligned}$$

which implies

2 $$\begin{aligned} \frac{n\log \alpha +\log \left( aC_1\right) }{\log p}<x<\frac{n\log \alpha +\log (aC_2)}{\log p}. \end{aligned}$$

We also have

3 $$\begin{aligned} \left| U_n-a\alpha ^n\right| <C_3|\alpha _2|^n, \end{aligned}$$

thus we have $x\asymp \frac{n\log \alpha }{\log p}$. In case that *U* satisfies also a strong dominant root condition we obtain a lower bound

4 $$\begin{aligned} C_4 |\alpha _2|^n< \left| U_n-a\alpha ^n\right| . \end{aligned}$$

Finally let us note that there exists $N_0$ such that $|U_n|$ is strictly increasing for $n\ge N_0$.

In the case of the Tribonacci sequence we can make these computations explicit. In particular, we have that

$$\begin{aligned} T_n=a\alpha ^n+b\beta ^n+{\bar{b}} \bar{\beta }^n. \end{aligned}$$

where $\alpha ,\beta$ und $\bar{\beta }$ are the roots of the characteristic Polynomial $X^3-X^2-X-1$, that is we have

$$\begin{aligned} \alpha\sim & {} 1.83929,\quad \beta \sim -0.419643 + 0.606291 i,\\ a= & {} \frac{5\alpha ^2-3\alpha -4}{22}\sim 0.336228 ,\quad b=\frac{5\beta ^2-3\beta -4}{22}\sim -0.168114 - 0.198324 i. \end{aligned}$$

Note that *a*, *b* und ${\bar{b}}$ are computed from solving the linear system

$$\begin{aligned} a\alpha ^0+b\beta ^0+{\bar{b}}\bar{\beta }^0&=0,\\ a\alpha ^1+b\beta ^1+{\bar{b}}\bar{\beta }^1&=1,\\ a\alpha ^2+b\beta ^2+{\bar{b}}\bar{\beta }^2&=1. \end{aligned}$$

Moreover, let us note that $\alpha \beta \bar{\beta }=-1$ and therefore $|\beta |=|\bar{\beta }|=|\alpha |^{-1/2}$. From these estimations for $\alpha$ and $\beta$ it is clear that $T_n\sim a\alpha ^n$. In particular we have that

5 $$\begin{aligned} 0.999 a\alpha ^n<T_n=p^x<1.001 a\alpha ^n \end{aligned}$$

provided that $n>8$. Note that (5) implies that $x<\frac{n\log \alpha }{\log p}$. Finally, let us note that the Tribonacci sequence $T_n$ is strictly increasing for $n\ge 2$.

In the case of the sequence *L* we find the explicit formula

$$\begin{aligned} L_n=\gamma ^n+1^n+\delta ^n, \end{aligned}$$

where

$$\begin{aligned} \gamma =\frac{1+\sqrt{5}}{2} \quad \text {and} \quad \delta =\frac{1-\sqrt{5}}{2}. \end{aligned}$$

Note that $\gamma \delta =-1$ and therefore $|\delta |=|\gamma |^{-1}$. Obviously *L* satisfies the strong dominant root condition but not the multiplicative independence relation. However, under the assumption that $n>10$ we obtain the estimates

6 $$\begin{aligned} \gamma ^n<L_n=p^x<1.009 \gamma ^n \end{aligned}$$

which imply

7 $$\begin{aligned} \frac{n\log \gamma }{\log p}<x < \frac{n\log \gamma +0.01}{\log p}. \end{aligned}$$

We also have the inequality

8 $$\begin{aligned} 0.99<\left| L_n-\gamma ^n\right| <1.01. \end{aligned}$$

Finally let us note that $L_n$ is strictly increasing for $n\ge 1$.

In this paper we will make extensive use of lower bounds for linear forms of logarithms of complex numbers. In particular we will use a result due to Matveev [10]. Let $\eta \ne 0$ be an algebraic number of degree *d* and let

$$\begin{aligned} a\left( X-\eta _1\right) \cdots \left( X-\eta _d\right) \in {\mathbb {Z}}[X] \end{aligned}$$

be the minimal polynomial of $\eta$. Then the absolute logarithmic Weil height is defined by

$$\begin{aligned} h(\eta )=\frac{1}{d} \left( \log |a|+\sum _{i=1}^d \max \{0,\log |\eta _i|\}\right) . \end{aligned}$$

In the case that $\eta$ is a rational number, say $\eta =P/Q \in {\mathbb {Q}}$ with *P*, *Q* integers such that $\gcd (P,Q)=1$, we have $h(P/Q)=\max \{\log |P|,\log |Q|\}$. With this basic notation we have the following result on lower bounds for linear forms in logarithms due to Matveev [10].

### Lemma 1

Denote by $\eta _1,\ldots ,\eta _m$ algebraic numbers, neither 0 nor 1, by $\log \eta _1$, $\ldots$, $\log \eta _m$ determinations of their logarithms, by *D* the degree over ${\mathbb {Q}}$ of the number field $K = {\mathbb {Q}}(\eta _1,\ldots ,\eta _m)$, and by $b_1,\ldots ,b_m$ rational integers. Furthermore let $\kappa =1$ if *K* is real and $\kappa =2$ otherwise. Choose

$$\begin{aligned} A_i\ge \max \left\{ D h\left( \eta _i\right) ,|\log \eta _i|\right\} \quad \left( 1\le i\le m\right) \end{aligned}$$

and

$$\begin{aligned} B=\max \left\{ 1,\max \left\{ |b_j| A_j /A_m: 1\le j \le m \right\} \right\} . \end{aligned}$$

Assume that $b_m\ne 0$ and $\log \eta _1,\ldots ,\log \eta _m$ are linearly independent over ${\mathbb {Z}}$. Then

$$\begin{aligned} \log |b_1\log \eta _1+\cdots +b_m \log \eta _m|\ge -C(m)C_0 W_0 D^2 \varOmega , \end{aligned}$$

with

$$\begin{aligned} \varOmega= & {} A_1\cdots A_m, \\ C(m)= & {} C(m,\kappa )= \frac{16}{m! \kappa } e^m \left( 2m +1+2 \kappa \right) (m+2)\left( 4(m+1)\right) ^{m+1} \left( \frac{1}{2} em\right) ^{\kappa }, \\ C_0= & {} \log \left( e^{4.4m+7}m^{5.5}D^2 \log (eD)\right) , \quad W_0=\log \left( 1.5eBD \log (eD)\right) . \end{aligned}$$

However, in the case that the number of logarithms is $m=2$ we have numerically rather good results due to Laurent [9]. In particular, we will use the following result:

### Lemma 2

Suppose that the numbers $\eta _1$, $\eta _2$, $\log \eta _1$, $\log \eta _2$ are real and positive and that $\eta _1$ and $\eta _2$ are multiplicatively independent. Then for positive integers $b_1$ und $b_2$ we have

$$\begin{aligned}&\log |b_1\log \eta _1 -b_2\log \eta _2| \\&\quad > - 17.9 D^4 \left( \max \left\{ \log b' + 0.38, 30/D, 1\right\} \right) ^2 \log A_1 \log A_2, \end{aligned}$$

where $$D=[{\mathbb {Q}}(\eta _1,\eta _2):{\mathbb {Q}}]$$,

$$\begin{aligned} A_i\ge \max \{h(\eta ),\log \eta /D,1/D\} \end{aligned}$$

for $$i=1,2$$ and

$$\begin{aligned} b'=\frac{b_1}{D\log A_2}+\frac{b_2}{D\log A_1}. \end{aligned}$$

In order to apply the results of Matveev and Laurent we have to know the heights of the relevant quantities and also have to ensure that they are multiplicatively independent. In the case of the Tribonacci sequence and the Lucas sequence added by one we can use Sage [14] to compute the heights of the relevant quantities and obtain

9 $$\begin{aligned} \begin{aligned} h(\alpha )&=h(\beta )=h(\bar{\beta })=0.20312595447866\dots<0.20313, \\ h(a)&=h(b)=h({\bar{b}})=1.2613965446394\dots<1.2614,\\ h(\gamma )&=h(\delta )=0.2406059125298\dots <0.2407 . \end{aligned} \end{aligned}$$

To ensure the multiplicative independence of $\alpha$, *a* and *p* in the case of the Tribonacci sequence and the Lucas sequence added by one the following lemma is helpful:

### Lemma 3

Let *p* be a prime, then $\alpha , a$ and *p* are multiplicatively independent and also $\gamma$ and *p* are multiplicatively independent.

### Proof

Since $\alpha$ and $\gamma$ are units and *p* is a prime it is obvious that $\alpha$ and *p* respectively $\gamma$ and *p* are multiplicatively independent. Thus it remains to show that $\alpha , a$ and *p* are multiplicatively independent.

Let us note that the splitting field $K={\mathbb {Q}}(\alpha ,\beta ,\bar{\beta })$ of the characteristic polynomial $X^3-X^2-X-1$ has class number 1. Moreover, 1/*a* is an algebraic integer with absolute Norm ${{\,\mathrm{N}\,}}_{K/{\mathbb {Q}}}(1/a)=2^4\cdot 11^2$. That is unless $p=2$ or $p=11$ the lemma is proved.

However, using Sage [14] we compute the following prime ideal factorizations

$$\begin{aligned} (2)&= {\mathfrak {p}}^3\\ (11)&= {\mathfrak {q}}_1^2 {\mathfrak {q}}_2^2 {\mathfrak {q}}_3^2\\ (1/a)&= {\mathfrak {p}}^2 {\mathfrak {q}}_1 {\mathfrak {q}}_2 \end{aligned}$$

which show that $\alpha ,a,2$ and 11 are multiplicatively independent and thus proving the lemma. $\square$

Also let us note the following helpful fact that can easily be proved using elementary calculus:

### Lemma 4

If $|x-1|<1/2$, then $|\log (x)|<3|x-1|/2$ and if $|y|<1/2$, then $\log (1+y)=y+\theta y^2$ for some $|\theta |\le 1$.

### Proof

Replace *x* by $1+y$ then the first statement is equivalent to the statement that $|y|<1/2$ implies $|\log (1+y)|<3|y|/2$. Thus assuming that $|y|<1/2$ we have

$$\begin{aligned} |\log (1+y)|&=\left| y-\frac{y^2}{2} \pm \dots \right| \\&\le |y|+\frac{|y|^2}{2}\left( 1+|y|+|y|^2+\dots \right) \\&=|y|\left( 1+\frac{|y|}{2}\cdot \frac{1}{1-|y|}\right) . \end{aligned}$$

From this inequality we easily deduce both statements. $\square$

In order to reduce the huge bounds coming from the applications of the results due to Matveev and Laurent we use continued fractions. In particular, we use the following two results:

### Lemma 5

Assume that $\mu$ is real and irrational and has the continued fraction expansion $\mu =[a_0;a_1,a_2,\dots ]$. Let $\ell$ be an integer and set $A=\max _{1\le j\le \ell }\{a_j\}$ and let $p_\ell /q_\ell$ be the $\ell$-th convergent to $\mu$, then

$$\begin{aligned} \frac{1}{(2 + A)q_\ell ^2} < \left| \mu - \frac{p}{q} \right| \end{aligned}$$

for any rational fraction *p*/*q* with $q\le q_\ell$.

### Proof

This follows from the inequality given in [2, page 47] combined with the best approximation property of continued fractions. $\square$

The second method is due to Baker and Davenport [3] for which we state a variant of this reduction method:

### Lemma 6

Given a Diophantine inequality of the form

10 $$\begin{aligned} |n\mu +\tau -x|<c_1\exp (-c_2 n), \end{aligned}$$

with positive constants $c_1,c_2$ and real numbers $\mu$ and $\tau$. Assume that $n<N$ and that there is a real number $\kappa >1$ such that there exists a convergent *p*/*q* to $\mu$ with

$$\begin{aligned} \Vert q \mu \Vert <\frac{1}{2 \kappa N} \quad \text {and} \quad \Vert q \tau \Vert >\frac{1}{\kappa }, \end{aligned}$$

where $\Vert \cdot \Vert$ denotes the distance to the nearest integer. Then we have

$$\begin{aligned} n\le \frac{\log (2 \kappa q c_1)}{c_2}. \end{aligned}$$

### Proof

We consider inequality (10) and multiply it by *q*. Then under our assumptions we obtain

11 $$\begin{aligned} \begin{aligned} c_1q\exp \left( -c_2 n\right)&>|qx+nq\mu +q\tau | \ge \left| \Vert q\tau \Vert -\Vert Nq\mu \Vert \right| \\&=\left| \Vert q\tau \Vert -N\Vert q\mu \Vert \right| > \frac{1}{2\kappa }. \end{aligned} \end{aligned}$$

Note that the equality in (11) holds since by assumption $N\Vert q\mu \Vert<\frac{1}{2\kappa }<\frac{1}{2}$ and therefore $N\Vert q\mu \Vert =\Vert Nq\mu \Vert$. Solving Inequality (11) for *n* yields the lemma. $\square$

## A first upper bound

In this section we will find absolute upper bounds for *x* and upper bounds for *n* in terms of *p* for solutions (*n*, *x*) to the Diophantine equations

12 $$\begin{aligned} U_n=p^x,\quad n\ge N_0 \end{aligned}$$

and

13 $$\begin{aligned} T_n=p^x,\quad n\ge 2 \end{aligned}$$

and

14 $$\begin{aligned} L_n=p^x,\quad n\ge 1. \end{aligned}$$

For a fixed prime *p* we can find such a bound by the standard procedure following the ideas of Shorey and Stewart [12] using Baker’s method.

### General method

Let us assume that that (*n*, *x*) is a solution to (12). Then Eq. (12) yields

$$\begin{aligned} \left| \frac{a\alpha ^{n}}{p^{x}}-1\right| \le \frac{C_3|\alpha _2^n|}{p^{x}}\le {\mathop {\overbrace{\frac{C_3}{|C_1a|}}}\limits ^{:=C_5}} \left| \frac{\alpha _2}{\alpha }\right| ^n. \end{aligned}$$

Taking logarithms yields

15 $$\begin{aligned} \varLambda =\left| n\log \alpha -x\log p+\log a\right| <\frac{3C_5}{2} \left| \frac{\alpha _2}{\alpha }\right| ^{n} \end{aligned}$$

Let us note that in the case that $\alpha$ and *a* are multiplicatively dependent we have integers $z_1$ and $z_2$ such that $\alpha ^{z_1}a^{z_2}=1$. Since $|\alpha |>1$ we have that $z_2\ne 0$ and therefore $\log a=\frac{-z_1 \log \alpha }{z_2}$. Thus we obtain in this case the inequality

16 $$\begin{aligned} \left| \left( nz_2-z_1\right) \log \alpha -x z_2\log p\right| <\frac{3z_2 C_5}{2} \left| \frac{\alpha _2}{\alpha }\right| ^{n}. \end{aligned}$$

We apply Matveev’s result depending on the multiplicative dependence of $\alpha$ and *a* to (15) with $m=3$ or to (16) with $m=2$. Therefore let us note that the heights of $\alpha$ and *a* are effective computable and that the height of *p* is $\log p$ and therefore (in the case that $m=3$) we have that $A_1,A_2$ are bounded by an absolute effective computable constant and $A_3=D\log p$, with $D=[{\mathbb {Q}}(a,\alpha ):{\mathbb {Q}}]$. Note that in the case that $m=2$ we have that $A_1$ is bounded by an effective computable constant and that $A_2=D\log p$. Thus we have in any case

$$\begin{aligned} B=\max \left\{ 1,\max \left\{ |b_j| A_j /A_m: 1\le j \le m \right\} \right\} \le C_6\frac{n}{\log p}. \end{aligned}$$

Let $S_0$ be the set of primes that divide the norm of $\alpha$, or divide the denominator of *a*, or divide the norm of *a*. Obviously the set $S_0$ is finite and for all primes $p\not \in S_0$ we have that $\alpha ,a$ and *p* are multiplicatively independent. Therefore let us assume that *p* is not a member of $S_0$, hence we obtain together with (15) the inequality

$$\begin{aligned} \log \left( \frac{3C_5}{2}\right) -n\log \left| \frac{\alpha }{\alpha _2}\right|>\log |\varLambda |>-C'_7 \log p \log \left( \frac{n}{\log p}\right) \end{aligned}$$

which yields

17 $$\begin{aligned} \frac{n}{\log p}< C_7 \log \left( \frac{n}{\log p}\right) \end{aligned}$$

provided that $$p\not \in S_0$$.

Note that in the case that $m=2$ similar considerations lead to the same conclusion and in particular to Inequality (17).

#### Lemma 7

Assume that (*n*, *x*) is a solution to (12) and that $p\not \in S_0$, then there exist effective computable constants $C_8$ and $C_9$ such that

$$\begin{aligned} n< C_8 \log p, \quad \text {and} \quad x <C_9. \end{aligned}$$

#### Proof

Inequality (17) implies an absolute upper bound for $\frac{n}{\log p}$. Since inequality (2) we have $x\asymp n \frac{\log |\alpha |}{\log p}$ and this immediately yields an absolute bound for *x* and also the bound for *n* stated in the lemma. $\square$

#### Remark 2

In Lemma 7 we basically reproved a result of Shorey and Stewart [12, Theorem 3]. But instead of using a result of Baker [1] on lower bounds for linear forms in logarithms we use the more explicit and easier to apply result due to Matveev [10].

#### Remark 3

Let us note that in the case that $\log \alpha$ and $\log a$ are linearly dependent over ${\mathbb {Q}}$ we may apply Laurent’s result (Lemma 2) and would obtain in concrete examples smaller numerical values for $C_7$ but the $\log \left( \frac{n}{\log p}\right)$ factor in Inequality (17) would turn into a $\log \left( \frac{n}{\log p}\right) ^2$ factor (see Sect. 3.3).

### Results for the Tribonacci sequence

Let us assume that (*n*, *x*) is a solution to (13) and in view of (5) we assume that $n\ge 10$. Then (13) implies

$$\begin{aligned} \left| \frac{a\alpha ^{n}}{p^{x}}-1\right| \le \frac{2|b||\beta |^{n}}{p^{x}}\le 1.55\, \alpha ^{-3n/2}. \end{aligned}$$

Taking logarithms on both sides yields

18 $$\begin{aligned} \left| n\log \alpha -x\log p+\log a\right| <2.33\, \alpha ^{-3n/2} \end{aligned}$$

We proceed to apply Matveev’s result. We set $D=3$, $\kappa =1$ and $m=3$.

$$\begin{aligned} \eta _1= & {} a, \quad \eta _2=\alpha , \quad \eta _3=p,\\ b_1= & {} 71, \quad b_2=n,\quad b_3=x. \end{aligned}$$

With this choice we have

$$\begin{aligned} A_1=3 h(a)<3.79, \quad A_2=3 h(\alpha )=3 \log \alpha <0.61, \quad A_3=3\log p. \end{aligned}$$

Furthermore we note that

19 $$\begin{aligned} p^x =T_n<1.001 a\alpha ^n<\alpha ^n. \end{aligned}$$

Thus $b_i|A_i|<3 n\log \alpha$ and therefore

$$\begin{aligned} B=\max \left\{ 1,\max \left\{ |b_j| A_j /A_m: 1\le j \le m \right\} \right\} <\max \left\{ 1,n\frac{\log \alpha }{\log p}\right\} =n\frac{\log \alpha }{\log p}. \end{aligned}$$

Finally we compute the quantities *C*(3) and $C_0$ numerically, put everything together and obtain

$$\begin{aligned} C(3)C_0W_0D^2\varOmega < 1.174 \cdot 10^{12}\log p \log (25.671 B). \end{aligned}$$

We apply the bound found above to inequality (18) and we obtain

$$\begin{aligned} 1.174 \cdot 10^{12} \log (25.671 B)>\frac{3n}{2}\cdot \frac{\log \alpha }{\log p}+ \frac{\log (2.33)}{\log p}. \end{aligned}$$

Let us write ${\tilde{B}}=25.671 B$, then we obtain the inequality

$$\begin{aligned} 3.06 \cdot 10^{10} \log {\tilde{B}}<{\tilde{B}}. \end{aligned}$$

Thus we obtain ${\tilde{B}} <8.411\cdot 10^{11}$, which yields $n<1.62\cdot 10^{11} \log p$. Therefore we have the following lemma.

#### Lemma 8

Let (*n*, *x*) be a solution to Diophantine Equation (13). Then we have $n<1.62\cdot 10^{11} \log p$ and $x<9.88\cdot 10^{10}$.

#### Proof

Only the bound for *x* is left to prove. However, since $x<\frac{n\log \alpha }{\log p}$ due to Inequality (19) the bound for *x* follows immediately. $\square$

#### Remark 4

For a fixed prime *p* it is easy to find explicit upper bounds for *n* using Lemma 8. Applying Baker–Davenport reduction to Inequality (18) we will find rather small explicit upper bounds for *n*, which are small enough to perform an explicit computer search for all solutions. This will be discussed in detail in Sect. 5.

### Results for the added by one Lucas sequence

Now, we will consider Diophantine Equation (14). In view of (6) we assume that $n>10$. In this case Diophantine Equation (14) can be written as

$$\begin{aligned} \gamma ^n+1+\delta ^n=p^x \end{aligned}$$

and we obtain due to (6) the inequality

$$\begin{aligned} \left| \frac{\gamma ^n}{p^x}-1\right|<\frac{1.009}{p^x}<1.009\,\gamma ^{-n}. \end{aligned}$$

Taking logarithms yields

20 $$\begin{aligned} |n\log \gamma -x\log p|<1.52\, \gamma ^{-n}. \end{aligned}$$

We apply Laurent’s result to this inequality. Therefore we note that with $K={\mathbb {Q}}(\gamma ,p)$ we have $D=[K:{\mathbb {Q}}]=2$. Let us set

$$\begin{aligned} \eta _1=\gamma ,\quad \eta _2=p,\quad b_1=n,\quad b_2=x. \end{aligned}$$

According to (9) we choose $A_1=0.2407$ and $A_2=\log p$. With this choice we obtain

$$\begin{aligned} b'=\frac{n}{2A_2}+\frac{x}{2A_1}<\frac{n}{\log p} \end{aligned}$$

due to inequality (7). If we assume that $\log b'\le 14.62$ we obtain $n< 2.24\cdot 10^6 \log p$ and due to (7) we also obtain $x<1.08\cdot 10^6$.

Therefore we will assume that $\log b'>14.62$ and Lemma 2 yields

$$\begin{aligned} n \log \gamma -\log (1.52)< 68.94 \log p \left( \log \left( 1.5 b'\right) \right) ^2. \end{aligned}$$

If we set ${\tilde{b}} =1.5 b'=\frac{1.5 n}{\log p}$ we obtain the inequality

$$\begin{aligned} {\tilde{b}}< 143.3 (\log {\tilde{b}})^2. \end{aligned}$$

Therefore we obtain that ${\tilde{b}}< 12822$ but this yields a contradiction to our assumption that $\log b'>14.62$. Therefore we have:

#### Lemma 9

Let (*n*, *x*) be a solution to Diophantine Equation (14). Then we have $n<2.24\cdot 10^6 \log p$ and $x<1.08\cdot 10^6$.

## Finding absolute upper bounds

### The multiplicative independence condition

Let us consider the Diophantine equation $U_n=p^x$, with $n\ge N_0$ and $p\not \in S_0$. Assume that *U* satisfies the multiplicative independence condition, i.e. $\log a$ and $\log \alpha$ are linearly independent over ${\mathbb {Q}}$. And in view of Theorem 1 we assume that there exist at least two solutions $(n_1,x_1)$ and $(n_2,x_2)$ with $n_1<n_2$. From (15) we obtain a system of inequalities:

$$\begin{aligned} \left| n_1\log \alpha -x_1\log p +\log a\right|&< \frac{3}{2} C_5 \left| \frac{\alpha _2}{\alpha }\right| ^{n_1},\\ \left| n_2\log \alpha -x_2\log p +\log a\right|&< \frac{3}{2} C_5 \left| \frac{\alpha _2}{\alpha }\right| ^{n_2}. \end{aligned}$$

We eliminate $\log p$ from this system by multiplying the first inequality by $x_1$ and the second by $x_2$ and subtracting them from each other. Then we obtain due to Lemma 7

21 $$\begin{aligned} \left| \varDelta \log \alpha + \varDelta _1\log a\right|< x_2 \frac{3}{2} C_5 \left| \frac{\alpha _2}{\alpha }\right| ^{n_1} +x_1 \frac{3}{2} C_5 \left| \frac{\alpha _2}{\alpha }\right| ^{n_2} < C_{10}\left| \frac{\alpha _2}{\alpha }\right| ^{n_1} \end{aligned}$$

with $\varDelta =n_1x_2-n_2x_1$ and $\varDelta _1=x_2-x_1$. Note that (21) implies that there exists an effectively computable constant $N_1$ such that for all $n_1\ge N_1$ we have

$$\begin{aligned} |\varDelta \log \alpha + \varDelta _1\log a|<1. \end{aligned}$$

Let us assume for the sake of the argument that $\max \{N_0,N_1\}\le n_1<n_2$. First we note that $\varDelta _1\ne 0$ since otherwise we would obtain $x_1=x_2$ and therefore also $n_1=n_2$ and the two solutions would be identical. Note that we assume that $N_0\le n_1<n_2$ and that $U_n$ is strictly increasing for $n\ge N_0$.

Let us assume for the moment that $\varDelta =0$. If $\varDelta =0$ we obtain

$$\begin{aligned} |\log a|<C_{10}\left| \frac{\alpha _2}{\alpha }\right| ^{n_1} \end{aligned}$$

which implies $$n_1\le C_{11}$$.

Now, let us consider the case that $\varDelta \ne 0$. Note that $\varDelta _1<C_9$ due to Lemma 7. Therefore inequality (21) yields

$$\begin{aligned} |\varDelta |\le \frac{\varDelta _1|\log a|+ C_{10}\left| \frac{\alpha _2}{\alpha }\right| ^{n_1}}{|\log \alpha |} < C_{12}. \end{aligned}$$

We may apply Matveev’s result (Lemma 1) or Laurent’s result (Lemma 2) to the linear form

$$\begin{aligned} \varLambda =\varDelta \log \alpha + \varDelta _1\log a \end{aligned}$$

and obtain that

$$\begin{aligned} -C_{13}<\log \left| \varDelta \log \alpha + \varDelta _1\log a\right| < -n_1 C_{14}, \end{aligned}$$

i.e. that $n_1<C_{15}$. Note that the last inequality holds for some Constant $C_{14}$ since we assume that $N_1\le n_1<n_2$. Therefore we have proved:

#### Proposition 1

Assume that $U_n$ satisfies the multiplicative independence condition and that the Diophantine equation $U_n=p^x$, with $n>\max \{N_0,N_1\}$ and $p\not \in S_0$ has at least two solution $(n_1,x_1)$ and $(n_2,x_2)$ with $\max \{N_0,N_1\}\le n_1<n_2$ and $x_1,x_2\ne 0$. Then there exists an effective computable constant $C_{15}$ such that $n_1<C_{15}$.

Let *S*(*U*) be the set of primes *p* such that $U_n$ is a power of *p* for some index *n* with $0\le n\le C_{15}$ together with the set of primes coming from the set $S_0$. Note that the set *S*(*U*) is finite and because $C_{15}$ is effectively computable the set *S*(*U*) is indeed effectively computable (at least in principal). With this notation we immediately deduce that the Diophantine equation $U_n=p^x$ has at most one solution with $x\ne 0$ if $p\not \in S(U)$. Thus we have proved Theorem 1 in this case.

Let us note that for a concrete given sequence *U* one can apply results from the theory of continued fractions (e.g. Lemma 5) to deduce from inequality (21) rather small upper bound for $n_1$. This method will be applied in the next subsection, when we deal with the Tribonacci sequence.

### Results for the Tribonacci sequence

We consider the Diophantine equation $T_n=p^x$. And in view of Theorem 2 we assume that there exist at least two solutions $(n_1,x_1)$ and $(n_2,x_2)$. If we can show that two such solutions with $4\le n_1<n_2$ do not exist we have proved Theorem 2. In view of Inequality (18) we assume that $9\le n_1<n_2$. With these assumptions Inequality (18) holds and we obtain a system of inequalities:

$$\begin{aligned} \left| n_1\log \alpha -x_1\log p +\log a\right|&< 2.33\, \alpha ^{-3n_1/2}\\ \left| n_2\log \alpha -x_2\log p +\log a\right|&< 2.33\, \alpha ^{-3n_2/2}. \end{aligned}$$

Multiplying the first inequality by $x_1$ and the second by $x_2$ and eliminating the $\log p$ term we obtain

22 $$\begin{aligned} \begin{aligned} \left| \varDelta \log \alpha + \varDelta _1\log a\right|&< x_2 2.33\, \alpha ^{-3n_1/2}+x_12.33 \alpha ^{-3n_2/2}\\&<2.33\, x_2\alpha ^{-3n_1/2} (1+1/\alpha )\\&<3.6\, x_2\alpha ^{-3n_1/2} \end{aligned} \end{aligned}$$

with $\varDelta =n_1x_2-n_2x_1$ and $\varDelta _1=x_2-x_1$. First, we note that $\varDelta _1\ne 0$ since otherwise $x_1=x_2$ and therefore also $n_1=n_2$ and the two solutions would be identical.

Let us assume for the moment that $\varDelta =0$. In this case we obtain

$$\begin{aligned} |\log a|<3.6 \, x_2 \alpha ^{-3 n_1/2}. \end{aligned}$$

Together with Lemma 8 we obtain in this case $n_1\le 30.3$.

Therefore we assume for the rest of this subsection that $\varDelta \ne 0$ and $n_1>30$. Using the upper bound for $x_2$ from Lemma 8 together with the assumption that $n_1\ge 31$ yields

$$\begin{aligned} \varDelta \le \frac{\varDelta _1|\log a|+3.6x_2\alpha ^{-3n_1/2}}{\log \alpha } < 1.77\cdot 10^{11}. \end{aligned}$$

And therefore we obtain

$$\begin{aligned} \left| \frac{\log \alpha }{-\log a}-\frac{\varDelta _1}{\varDelta }\right|<3.6 \frac{x_2}{-|\varDelta |\log a}\alpha ^{-3n_1/2}<3.27\cdot 10^{11}\alpha ^{-3n_1/2}. \end{aligned}$$

Since continued fractions are the best approximations we compute the continued fractions of $\mu =\frac{\log \alpha }{-\log a}$ and notice that the 32-nd convergent $p_{32}/q_{32}=\frac{121491924785}{217306873051}$ is the first convergent such that its denominator is larger than $1.77\cdot 10^{11}$. Moreover, we compute $A=15$ and therefore we obtain by Lemma 5 the inequality

$$\begin{aligned} 1.24\cdot 10^{-24}<\frac{1}{(A+2)q_{32}^2}< \left| \frac{\log \alpha }{-\log a}-\frac{\varDelta _1}{\varDelta }\right| <3.27\cdot 10^{11}\alpha ^{-3n_1/2} \end{aligned}$$

which yields $$n_1\le 89.7$$.

If we factor the first 89 Tribonacci numbers and only take those into account which are primes or prime powers we obtain

#### Proposition 2

If the Diophantine equation $T_n=p^x$ has at least two solutions $(n_1,x_1)$ and $(n_2,x_2)$ with $0\le n_1<n_2$ and $x\ne 0$, then

$$\begin{aligned} \left( n_1,x_1,p\right)&= (3,1,2),(4,2,2),(5,1,7),(6,1,13),(9,4,3),(10,1,149),\\&\quad (17,2,103)(86,1,19341322569415713958901), \end{aligned}$$

if a second solution exists at all.

#### Remark 5

The proof of Theorem 2 is complete, if we can show that the Diophantine equation $T_n=p^x$, with $n\ge 3$ has at most one solution if $p=3,7,13,103,149$ or $p=19341322569415713958901$ and at most two solutions if $p=2$. As already mentioned this can be done by using the Baker–Davenport reduction which will be discussed in Sect. 5.

### The strong dominant root case

Since the case that *a* and $\alpha$ are multiplicatively independent has been resolved, we may assume that *a* and $\alpha$ are multiplicatively dependent, that is we fix integers $z_1$ and $z_2$ such that $\alpha ^{z_1}a^{z_2}=1$ and $(z_1,z_2)\ne (0,0)$. That is

$$\begin{aligned} n\log \alpha +\log a=\frac{z_2n-z_1}{z_2}\log \alpha . \end{aligned}$$

Let us also note that since we assume a strong dominant root condition we have instead of (15) the inequality

$$\begin{aligned} C_{16} \left| \frac{\alpha _2}{\alpha }\right| ^{n}<\left| {\tilde{n}}\log \alpha -xz_2\log p\right| <2C_5 z_2 \left| \frac{\alpha _2}{\alpha }\right| ^{n}=C_{17}\left| \frac{\alpha _2}{\alpha }\right| ^{n}, \end{aligned}$$

with $${\tilde{n}}=z_2n-z_1$$.

Before we proceed with the proof of Theorem 1 in the strong dominant root case we prove the following lemma.

#### Lemma 10

There exists an effective computable constant $N_1$ such that $C_{16}$ and $C_{17}$ can be chosen such that $\frac{C_{17}}{C_{16}}<\left| \frac{\alpha }{\alpha _2}\right|$, provided that $n\ge N_1$.

#### Proof

Since $p^x=U_n$ there is a positive constant $A_3$ such that

$$\begin{aligned} p^x=a\alpha ^n+a_2\alpha _2^n+\theta A_3 |\alpha _3|^n \end{aligned}$$

for some $|\theta |\le 1$. With these notations we compute

$$\begin{aligned} \varLambda&=|x\log p-n\log \alpha -\log a|=\left| \log \left( \frac{p^x}{a\alpha ^n}\right) \right| \\&\le \left| \log \left( 1+\frac{a_2 \alpha _2^n}{a\alpha ^n}+\frac{A_3 |\alpha _3|^n}{a\alpha ^n}\right) \right| . \end{aligned}$$

Now, we find an explicit upper bound for $\varLambda$, provided that *n* is chosen large enough such that $\frac{a_2 \alpha _2^n}{a\alpha ^n}+\frac{A_3 |\alpha _3|^n}{a\alpha ^n}<\frac{1}{2}$ holds. According to Lemma 4 we have

$$\begin{aligned} \varLambda&\le \left| \frac{a_2 \alpha _2^n}{a\alpha ^n}\right| +\left| \frac{A_3 \alpha _3^n}{a\alpha ^n}\right| +\left( \left| \frac{a_2 \alpha _2^n}{a\alpha ^n}\right| +\left| \frac{A_3 \alpha _3^n}{a\alpha ^n}\right| \right) ^2\\&=\left| \frac{a_2 \alpha _2^n}{a\alpha ^n}\right| \cdot \left( 1+\left| \frac{A_3 \alpha _3^n}{a_2\alpha _2^n}\right| +\left| \frac{a_2 \alpha _2^n}{a\alpha ^n}\right| + 2\left| \frac{A_3 \alpha _3^n}{a\alpha ^n}\right| +\left| \frac{A_3^2 \alpha _3^{2n}}{a_2 a\alpha ^n\alpha _2^n}\right| \right) \\&<\left| \frac{a_2 \alpha _2^n}{a\alpha ^n}\right| \cdot \left( 1+\left( \left| \frac{A_3}{a_2}\right| +\left| \frac{a_2}{a}\right| +2\left| \frac{A_3}{a}\right| +\left| \frac{A_3^2}{aa_2}\right| \right) \max \left\{ \left| \frac{\alpha _2}{\alpha }\right| ,\left| \frac{\alpha _3}{\alpha _2}\right| \right\} ^n\right) \end{aligned}$$

Similarly we obtain

$$\begin{aligned} \varLambda >\left| \frac{a_2 \alpha _2^n}{a\alpha ^n}\right| \cdot \left( 1-\left( \left| \frac{A_3}{a_2}\right| +\left| \frac{a_2}{a}\right| +2\left| \frac{A_3}{a}\right| +\left| \frac{A_3^2}{aa_2}\right| \right) \max \left\{ \left| \frac{\alpha _2}{\alpha }\right| ,\left| \frac{\alpha _3}{\alpha _2}\right| \right\} ^n\right) \end{aligned}$$

Under the assumption that $n\ge N$ we can choose $C_{16}$ and $C_{17}$ such that

23 $$\begin{aligned} \frac{C_{17}}{C_{16}}\le \frac{1+{\tilde{c}} \varGamma ^N}{1-{\tilde{c}} \varGamma ^N} \end{aligned}$$

where we write $\varGamma =\max \left\{ \left| \frac{\alpha _2}{\alpha }\right| ,\left| \frac{\alpha _3}{\alpha _2}\right| \right\}$ and ${\tilde{c}}=\left| \frac{A_3}{a_2}\right| +\left| \frac{a_2}{a}\right| +2\left| \frac{A_3}{a}\right| +\left| \frac{A_3^2}{aa_2}\right|$. Since the right hand side of (23) converges to 1 as $N\rightarrow \infty$ we obtain the content of the lemma. $\square$

Let us assume that $U_n=p^x$ has two solutions $(n_1,x_1)$ and $(n_2,x_2)$ with $x_1,x_2> 0$ and $N_0\le n_1<n_2$. This yields the following system of inequalities:

$$\begin{aligned} C_{16} \left| \frac{\alpha _2}{\alpha }\right| ^{n_1}<\left| {\tilde{n}}_1\log \alpha -x_1z_2\log p\right|&< C_{17} \left| \frac{\alpha _2}{\alpha }\right| ^{n_1}\\ C_{16} \left| \frac{\alpha _2}{\alpha }\right| ^{n_2}<\left| {\tilde{n}}_2\log \alpha -x_2z_2\log p\right|&< C_{17} \left| \frac{\alpha _2}{\alpha }\right| ^{n_2}. \end{aligned}$$

Eliminating $\log p$ yields similar as in the case that *a* and $\alpha$ are multiplicatively independent the inequality

$$\begin{aligned} C_{16} x_2\left| \frac{\alpha _2}{\alpha }\right| ^{n_1}-C_{17} x_1\left| \frac{\alpha _2}{\alpha }\right| ^{n_2}<|\varDelta \log \alpha | <C_{18}\left| \frac{\alpha _2}{\alpha }\right| ^{n_1}, \end{aligned}$$

with $\varDelta ={\tilde{n}}_1 x_2-{\tilde{n}}_2x_1$. Let us note that if we assume that $n_1\ge N_1$ where $N_1$ is the bound from Lemma  10 we obtain

$$\begin{aligned} \frac{C_{17}x_1}{C_{16}x_2}<\frac{C_{17}}{C_{16}}<\left| \frac{\alpha }{\alpha _2}\right| \le \left| \frac{\alpha }{\alpha _2}\right| ^{n_2-n_1}=\frac{\left| \frac{\alpha _2}{\alpha }\right| ^{n_1}}{\left| \frac{\alpha _2}{\alpha }\right| ^{n_2}}, \end{aligned}$$

which implies

$$\begin{aligned} C_{16} x_2\left| \frac{\alpha _2}{\alpha }\right| ^{n_1}-C_{17} x_1\left| \frac{\alpha _2}{\alpha }\right| ^{n_2}>0. \end{aligned}$$

Therefore we deduce that $\varDelta ={\tilde{n}}_1 x_2-{\tilde{n}}_2x_1\ne 0$, provided that $n\ge N_1$. Therefore we obtain

$$\begin{aligned} |\log \alpha | <C_{18}\left| \frac{\alpha _2}{\alpha }\right| ^{n_1} \end{aligned}$$

which yields $n_1<C_{19}$ and we obtain:

#### Proposition 3

Assume that $U_n$ satisfies the strong dominant root condition and that the Diophantine equation $U_n=p^x$ with $n>N_0$ and $p\not \in S_0$ has at least two solutions $(n_1,x_1)$ and $(n_2,x_2)$ with $n_1<n_2$ and $x_1,x_2\ne 0$. Then there exists an effective computable constant $C_{19}$ such that $n_1<C_{19}$.

As in the case that the multiplicative independence condition holds we deduce now Theorem 1 in this case. Thus Theorem 1 is completely proved.

### Results for the added by one Lucas sequence

Let us assume that the Diophantine equation $L_n=p^x$ has at least two solutions $(n_1,x_1)$ and $(n_2,x_2)$. We follow the arguments of the strong dominant root case where $\log \alpha$ and $\log a$ are linearly dependent. First we compute a lower bound $N_1$ for which Lemma 10 holds. Therefore let us note that we have $a=a_2=1$ and that we can choose $A_3=1$. Thus we have ${\tilde{c}}=5$. Moreover, we have $\alpha =\frac{1+\sqrt{5}}{2}$, $\alpha _2=1$ and $\alpha _3=\frac{1-\sqrt{5}}{2}$ and we can choose $\varGamma =\frac{-1+\sqrt{5}}{2}=\gamma ^{-1}$ in (23). Therefore we have to find a bound $N_1$ such that

$$\begin{aligned} \frac{1+\sqrt{5}}{2}>\frac{1+5\gamma ^{-n}}{1-5\gamma ^{-n}} \end{aligned}$$

for all $n\ge N_1$. By an explicit computation we obtain that $N_1=7$ is sufficient.

Now we consider the following system of inequalities under the assumption that $n_1> 7$.

$$\begin{aligned} \left| n_1\log \gamma -x_1\log p\right|&< 1.52 \gamma ^{-n_1}\\ \left| n_2\log \gamma -x_2\log p\right|&< 1.52 \gamma ^{-n_2}. \end{aligned}$$

By eliminating $\log p$ we obtain

$$\begin{aligned} |\varDelta \log \gamma |<1.52 x_2 \gamma ^{-n_1}+1.52 x_1\gamma ^{-n_2}<3.29\cdot 10^6 \gamma ^{-n_1}. \end{aligned}$$

Since the computations of the previous subsection we may assume that $\varDelta \ne 0$ and obtain

$$\begin{aligned} \gamma ^{n_1}< 6.83 \cdot 10^6 \end{aligned}$$

and therefore we have $n_1<32.71$.

If we factor the first 32 members of the sequence *L* and only consider those which are primes or prime powers we obtain

#### Proposition 4

If the Diophantine equation $L_n=p^x$ has at least two solutions $(n_1,x_1)$ and $(n_2,x_2)$ with $0\le n_1<n_2$ and $x\ne 0$, then

$$\begin{aligned} \left( n_1,x_1,p\right) =(0,1,3),(1,1,2),(2,2,2),(3,1,5),(4,3,2),(6,1,19),(18,1,5779), \end{aligned}$$

if a second solution exists at all.

## Reduction of the upper bounds for a fixed prime

The purpose of this last section is to prove Theorems 2 and 3 .

### Application of the Baker–Davenport reduction method

We consider the Diophantine equation $T_n=p^x$ with $x\ne 0$ and

$$\begin{aligned} p\in {\mathcal {P}}=\{2,3,7,13,103,149,p_0\} \end{aligned}$$

with $p_0=19341322569415713958901$ and $n<1.62\cdot 10^{11} \log p$. In particular we consider Inequality (18) and divide it through $\log p$ and obtain

$$\begin{aligned} \left| n\frac{\log \alpha }{\log p}+\frac{\log a}{\log p} -x\right| <\frac{2.33}{\log p}\exp \left( -\frac{3\log \alpha }{2} n\right) . \end{aligned}$$

Thus we apply Lemma 6 with

$$\begin{aligned} \mu =\frac{\log \alpha }{\log p},\quad \tau =\frac{\log a}{\log p},\quad c_1=\frac{2.33}{\log p},\quad c_2=\frac{3\log \alpha }{2},\quad N=1.62\cdot 10^{11} \log p \end{aligned}$$

for each $p\in {\mathcal {P}}$. In each case we obtain a new bound for *n*. In Table 1 we indicate for which prime *p* the $\ell$-th convergent yields a new upper bound *B* for *n*.

**Table 1:** New bounds $n\le B$ after the Baker–Davenport reduction

*p*	$\ell$	*B*	*p*	$\ell$	*B*
2	20	33.18	103	33	31.8
3	21	35.65	149	24	33.07
7	23	28.44	$p_0$	58	89.27
13	34	32.12			

Thus we conclude that if $T_n=p^x$ has two solutions $(n_1,x_1)$ and $(n_2,x_2)$ with $x_1,x_2\ne 0$ and $n_1<n_2$, then $n_2\le 89$. However a quick computer search among the first 89 Tribonacci numbers shows that only in the case that $p=2$ we have two solutions namely $T_3=2$ and $T_4=4$. Thus the proof of Theorem 2 is complete.

### Using continued fractions

Now we consider the Diophantine equation $L_n=p^x$ with $x\ne 0$ and $p\in {\mathcal {P}}=\{2,3,5,19,5779\}$ and $x<1.08 \cdot 10^6$. In particular we consider the inequality

$$\begin{aligned} \left| \frac{n}{x} -\frac{\log p}{\log \gamma }\right| <3.54 \gamma ^{-n} \end{aligned}$$

which we deduce from (20). We apply Lemma 5 to this inequality with $\mu = \frac{\log p}{\log \gamma }$ and the smallest convergent $p_\ell /q_\ell$ to $\mu$ such that $q_\ell >1.08 \cdot 10^6$. For the continued fraction to $\mu$ we compute according to Lemma 5 the quantity *A* and obtain

$$\begin{aligned} \frac{1}{(2 + A)q_\ell ^2}<\left| \frac{n}{x} -\frac{\log p}{\log \gamma }\right| <3.54\gamma ^{-n} \end{aligned}$$

and therefore we obtain

$$\begin{aligned} n<B=\frac{\log \left( 3.54 (2 + A)q_\ell ^2\right) }{\log \gamma }. \end{aligned}$$

If we compute all the quantities for all $p\in {\mathcal {P}}$ we obtain new bounds for *n*. In Table 2 we give the relevant quantities for each prime $p\in {\mathcal {P}}$.

**Table 2:** Bounds for *n* after the application of Lemma 5

*p*	*A*	$\ell$	*B*	*p*	*A*	$\ell$	*B*
2	11	15	71.53	19	26	12	72.14
3	49	16	71.87	5779	2780	5	84.88
5	20	13	66.97				

This implies that in any case $n_2\le 84$. Thus we compute the first 84 members of *L* and check whether they are a power of any of the primes $p\in {\mathcal {P}}$. A quick computer search yields that the only case for which more than one solution exists is $p=2$. In this case we have the three solutions $L_1=2$, $L_2=4$ and $L_4=8$. Thus Theorem 3 is proved.
